# Optimization and Characterization of Preceramic Inks for Direct Ink Writing of Ceramic Matrix Composite Structures

**DOI:** 10.3390/ma11040515

**Published:** 2018-03-28

**Authors:** Giorgia Franchin, Halide Selin Maden, Larissa Wahl, Andrea Baliello, Marco Pasetto, Paolo Colombo

**Affiliations:** 1 Department of Industrial Engineering, University of Padova, 35131 Padova, Italy; halideselinmaden@anadolu.edu.tr (H.S.M.); larissa.wahl@fau.de (L.W.); paolo.colombo@unipd.it (P.C.); 2 Department of Civil, Environmental and Architectural Engineering, University of Padova, 35131 Padova, Italy; andrea.baliello@dicea.unipd.it (A.B.); marco.pasetto@dicea.unipd.it (M.P.); 3 Materials Science and Engineering, The University of Pennsylvania, State College, PA 16801, USA

**Keywords:** CMC, additive manufacturing, preceramic polymers

## Abstract

In a previous work, an ink based on a preceramic polymer, SiC fillers, and chopped carbon fibers was proposed for the production of Ceramic Matrix Composite (CMC) structures by Direct Ink Writing (DIW) and subsequent pyrolysis. Thanks to the shear stresses generated at the nozzle tip during extrusion, carbon fibers can be aligned along the printing direction. Fumed silica was added to the ink in order to enhance its rheological properties; however, the printed structures still showed some deformation in the Z direction. In this work, a second ink was successfully developed to limit deformation and at the same time avoid the addition of fumed silica, which limited the potential temperature of application of the composites. Instead, the positive role of the preceramic polymer on the ink rheology was exploited by increasing its concentration in the ink. Rheological characterization carried out on both inks confirmed that they possessed Bingham shear thinning behavior and fast viscosity recovery. Single filaments with different diameters (~310 µm and ~460 µm) were produced with the latter ink by DIW and subsequent pyrolysis. Tested under a four-point flexural test, the filaments showed a mean flexural strength above 30 MPa, graceful failure, and fiber pull-out. The results of this work suggest that CMC components can effectively be fabricated via DIW of a preceramic ink with embedded short fibers; the preceramic polymer is able to provide the desired rheology for the process and to develop a dense matrix capable of incorporating both fibers and ceramic particles, whereas the fibers addition contributes to an increase of the fracture toughness of the material and to the development of a graceful failure mode.

## 1. Introduction

Preceramic polymers are a class of inorganic polymers that can be converted into a ceramic with high yield, and materials resulting from this process are termed polymer-derived ceramics (PDC). Most preceramic polymers contain silicon in the backbone and, depending on the specific precursor, ceramics such as SiC, Si_3_N_4_, SiCN, SiOC, or SiOCN can be produced (the latter ones can be obtained only by this molecular route). PDCs can be manufactured at lower temperatures (1000–1300 °C) compared to classical powder technology, and exhibit superior oxidation resistance and excellent thermo-mechanical properties up to and above 1500 °C [1]. Moreover, they can also possess functional properties such as piezo-resistivity, electrical conductivity, luminescence, high chemical durability, and biocompatibility [2,3,4,5,6,7]. 

Their greatest advantage is that they can be processed in the liquid, solid, or molten state using a wide range of conventional polymer-forming technologies, such as infiltration, extrusion, injection molding, blowing, melt- and electro-spinning, etc., overcoming the general shaping limitations of the traditional ceramic processing. Indeed, this enables them to be used in different Additive Manufacturing (AM) technologies, as demonstrated by publications concerning ink-jet printing [8], selective laser curing [9], powder-bed 3D printing [10,11], direct ink writing [12,13], and stereolithography [14,15,16,17].

Preceramic polymers are also widely employed for the manufacturing of ceramic matrix composites (CMCs), non-brittle refractory materials meant to be employed in severe environments. The superior mechanical properties of CMCs are provided by the presence of high strength, high modulus ceramic fibers of small diameter as a reinforcement [18].

The process, known as liquid polymer infiltration (LPI) or polymer infiltration and pyrolysis (PIP), requires the preceramic polymers to be infiltrated into a fiber preform. During the subsequent heat treatment, the polymers are pyrolyzed and the final ceramic structure is obtained. Typically, several re-infiltration steps are required to densify the matrix and fill the cracks and porosity that develop during pyrolysis. Components can be machined to near-net shape before full densification and no extensive final machining is needed. On the other hand, shapes that can be produced by infiltration are rather conventional and do not allow for the fabrication of lattices with internal complexity and voids.

PIP processes are mainly suited for the production of continuous fiber-reinforced ceramics; short fiber-reinforced CMCs, however, combine a generally lower price with good mechanical properties and the possibility of using a wider range of manufacturing processes like hot pressing, tape casting, injection molding, or extrusion. They are employed for advanced applications such as disk brakes and pads for high-end cars, thanks to their low wear rates and high coefficients of friction [19,20].

A few works have recently been published on the development of CMCs via AM routes [21,22,23]; most research has so far been focused on AM of polymer matrix composites, thanks to the presence of more established technologies for polymers [24,25,26,27]. In particular, the extrusion of composite inks with direct ink writing (DIW) methods showed that it is possible to align short fibers and other directional reinforcements along the printing direction simply by exploiting the shear stresses generated at the nozzle tip [28,29,30].

The suitability of a preceramic ink containing chopped carbon fibers for the production of CMC components with complex shapes through DIW and subsequent pyrolysis was demonstrated in a previous work [31]; as postulated, the carbon fibers in the extruded filaments appeared to be effectively aligned along the printing direction. Although the produced samples possessed complex shapes and open porosity in the X, Y, and Z directions, they were not fully able to withstand their weight; moreover, the use of fumed silica as a rheological additive could potentially limit their use at high temperatures (whereas a multilayer environmental barrier coating on the composite could easily seal it and overcome the oxidation issues related to the use of carbon fibers) [18,32]. In this paper, the rheological behavior of the ink was characterized in detail; moreover, a new ink was formulated in order to better retain the samples’ shape and to avoid the use of silica-based additives. The new composition was used to produce single filaments which were tested under a four-point bending test in order to understand the mechanism of failure for the resulting composite.

## 2. Materials and Methods 

### 2.1. List of Reagents

- Preceramic polymer: poly(methyl-silsesquioxane) Silres MK (Wacker Chemie AG, Nünchritz, Germany);
- Solvent: isopropanol;
- Inert filler: SiC powder Starceram S Grade UF 10 (H.C. Starck Ceramics GmbH, Selb, Germany, d_50_ = 0.7 µm);
- Dispersant: BYK 180 (BYK-Chemie GmbH, Wesel, Germany);
- Reinforcement: chopped, uncoated carbon fibers MF100 (Ferrari Carbon Srl, Milano, Italy) with a length of 100 µm and a thickness of 7.5 µm;
- Rheology modifier: hydrophobic fumed silica Aerosil R106 (Evonik, Essen, Germany; specific surface area = 220–280 m^2^/g);
- Crosslinking catalyst: Geniosil GF91 (Wacker Chemie AG, Nünchritz, Germany).

### 2.2. Preparation of Ink A

The preparation of the first ink involved several steps:

1. MK dissolution into isopropanol in a 70:30 weight ratio at room temperature via ball milling for ~8 h (higher volumes of the solution were prepared in advance and stored at room temperature in a sealed container);
2. Addition of SiC powder and dispersant and mechanical stirring at low speed (<200 rpm) to avoid air entrapment. The SiC powder was added to reduce the formation of cracks upon pyrolysis generated during the polymer-to-ceramic conversion of the preceramic polymer [31]. As the preceramic polymer converts to SiOC in inert atmosphere with a ceramic yield of ~85 wt %, the SiC addition was calculated as a 50:50 weight ratio on the SiOC amount. The dispersant was employed to assure a good dispersion of the powder in the ink.
3. Dispersion of carbon fibers (~17 vol % of the ceramized CMC) via mechanical stirring at low speed for ~30 min;
4. Addition of fumed silica under mechanical stirring at low speed;
5. Addition of GF91 (0.5 wt % ratio on MK) under mechanical stirring at low speed for 10 min to catalyze the crosslinking of the preceramic polymer.

### 2.3. Preparation of Ink B

The preparation of the second ink involved the same reagents (except for the fumed silica, which was not added to the mixture) and similar processing steps:

1. MK dissolution into isopropanol;
2. Addition of SiC powder and dispersant; the SiC content was slightly decreased (45:55 weight ratio for the SiOC amount);
3. Dispersion of carbon fibers (~21 vol % of the ceramized CMC);
4. Controlled evaporation of the solvent achieved by stirring the ink on a hot plate at 100 °C (Ink B) until a thick gel was achieved;
5. Addition of GF91.

Instead of adding the fumed silica as a gel former, the control of the rheology was achieved in this ink by decreasing the content of the solvent and thus exploiting the presence of the polymer.

The two ink formulations are provided in Table 1. In the second ink, it was possible to slightly increase the carbon fiber content; the SiC content was decreased accordingly. 

### 2.4. Rheological Properties

The rheological behavior of the two inks was conducted using a rotational rheometer (MCR 302, Anton Paar, Graz, Austria) equipped with a 50-mm diameter plate-plate geometry, with a set temperature of 20 °C and a gap of 1 mm.

The rheology of the inks is the key factor for the DIW process, and is crucial for the fabrication of geometries able to retain their shape; in fact, the material has to bear its own weight with minimal deformation after being printed. This objective can be achieved using a shear thinning behavior with an initial yield stress (Bingham pseudoplastic behavior): when a shear stress is applied, the viscosity of the ink decreases so that it can flow under moderate pressures; on the other hand, once the stress is released, the viscosity increases again quickly and the printed structure freezes in place.

Several experiments were conducted in order to fully characterize our mixtures. Specifically,

1. Working time: constant shear rate of 0.01 1/s over 2 h;
2. Steady rate sweep: shear rate increasing from 0.01 to 10 1/s;
3. Dynamic strain sweep: strain varying from 0.001 to 100% with a frequency of 1 Hz;
4. Dynamic frequency sweep: frequency varying from 0.1 to 100 Hz with a strain of 5%;
5. Viscosity recovery, in two steps: first, a shear rate of 10 1/s for 60 s was applied, followed by the application of a controlled shear stress of 15 Pa for 120 s in order to measure the recovery of viscosity. The shear rate needed to be high enough to overcome the initial yield stress of the ink; in the second stage, the shear stress needed to be lower than the yield stress to allow for recovery. Values were chosen according to the results of the first two tests.

Rheological tests were performed on freshly prepared inks and were repeated for a minimum of two measurements; mean values are plotted.

### 2.5. Extrusion and Pyrolysis of Single Filaments

The production of single filament components mimicked the conventional DIW process; a commercial fused deposition modeling printer for polymeric materials (Delta Wasp 2040 Turbo, Wasproject, Massa Lombarda, Italy) was equipped with a pressurized paste extrusion system which can mount conical nozzles of various sizes (Nordson Italia S.p.a., Segrate, Italy), ranging from 100 to 1500 µm. 

**Figure 1:**
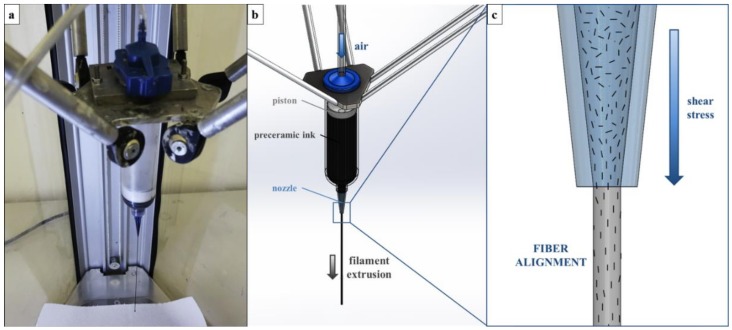
Overview of the filament production process: (**a**) filaments extruded with a 410-µm nozzle; (**b**) schematics of the extrusion process; (**c**) magnification of the nozzle tip, showing the fiber alignment in the extrusion direction driven by the shear stress generated by the process (fibers are drawn to scale).

An overview and schematics of the process are shown in Figure 1. As the ink is pushed through a thin nozzle, shear stresses are generated at the nozzle tip which are able to rotate the carbon fibers and orient them along the printing direction (see Figure 1c).

The filaments were produced by continuous extrusion in the vertical direction using Ink B. A pressure of ~2 bar was applied to the extrusion system. Samples were produced using two different nozzle tips, one with an inner diameter of 410 µm and the other with a diameter of 580 µm.

After printing, the samples were hung (to avoid deformation), dried at room temperature overnight, and then heat-treated at a rate of 1 °C/min to 1000 °C (1 h dwelling time) under flowing nitrogen (99.99%). Finally, the filaments were cut into 20-mm long rods for mechanical testing.

### 2.6. Physical Characterization of the Filaments

The morphology of the samples was investigated by stereomicroscopy (STEMI 2000-C, Carl Zeiss AG, Oberkochen, Germany) and scanning electron microscopy (ESEM, Quanta 200, FEI, Hillsboro, OR, USA). The shrinkage of the samples was calculated after measurement of the diameters of the green and pyrolized samples in the collected images.

### 2.7. Four-Point Bending Test

The ceramized samples were tested at room temperature with four-point bending equipment using an Instron 1121 UTM (Instron, Danvers, MA, USA) at cross-head speed of 0.5 mm/min with a support span L (mm) of 13 mm.

**Figure 2:**
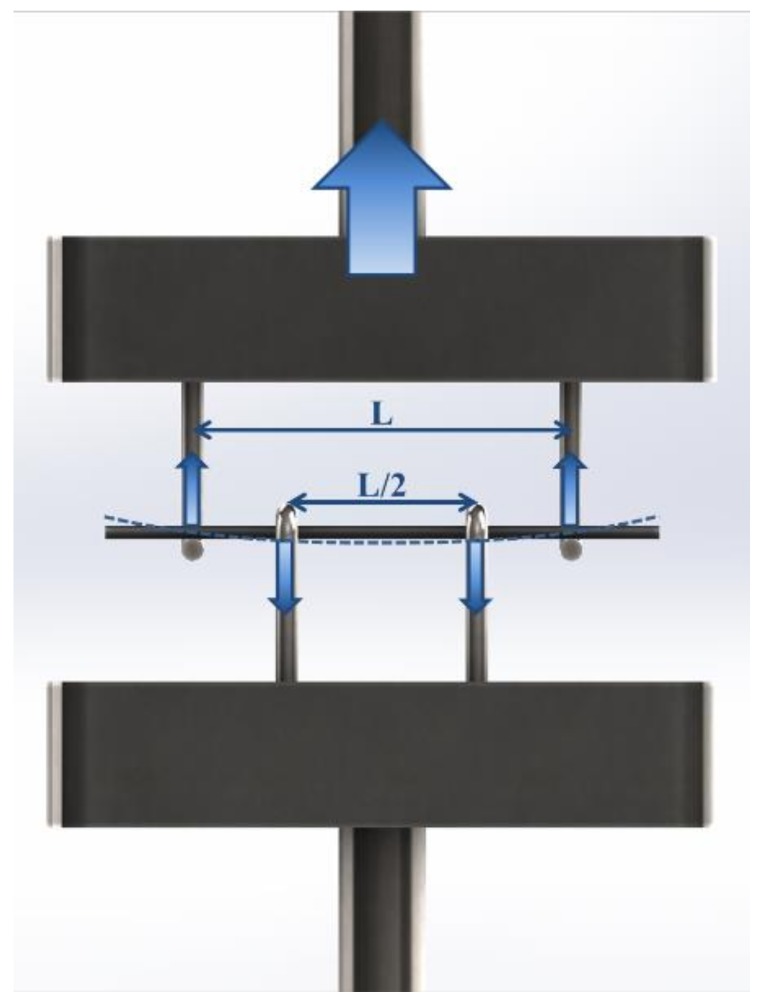
Schematic of the four-point bending test custom equipment and operation on a pyrolyzed filament sample with a diameter of 460 µm and a length of 20 mm.

The four-point bending equipment was custom designed and requires the machine to operate in tensile mode; a 500 gf load cell with high sensibility could therefore be employed. Schematics of the equipment and its operation are shown in Figure 2.

The flexural strength $\sigma_{f}$ (MPa) was obtained from the maximum load *F* (N) recorded during the test according to the equation:

(1) $$\sigma_{f}=\frac{Mc}{I}$$

where *M* (N·mm) is the applied moment, *c* (mm) is the distance from the neutral axis, and *I* (mm^4^) is the moment of inertia of the cross-section perpendicular to the neutral axis [33].

The loading span *l* (mm) was equal to *L*/2; therefore, the applied moment can be expressed as follows:

(2) $$M=\frac{F}{2}\frac{L-l}{2}=\frac{FL}{8}$$

For a beam with circular cross-section:

(3) $$I=\frac{\pi d^{4}}{64},$$

(4) $$c=\frac{d}{2},$$

where *d* (mm) is the diameter of the tested filament [33].

The flexural strength can be calculated as follows:

(5) $$\sigma_{f}=\frac{FL}{8}\frac{d}{2}\frac{64}{\pi d^{4}}=\frac{4FL}{\pi d^{3}},$$

The results were reported as mean ± standard deviation measured over 10 samples.

## 3. Results and Discussion

### 3.1. Rheological Properties of the Inks

The first objective of this rheological characterization was to assess the behavior of the ink developed in the first phases of this project and published in our previous work [31]. In this case, the Bingham shear thinning behavior was enhanced by the addition of fumed silica as a reversible gel former. The second objective was to validate the second ink, developed with the goal of eliminating the addition of fumed silica, in which the polymeric nature of the preceramic precursor was exploited by reducing the amount of solvent and taking advantage of its fast evaporation and of the polymeric nature of the preceramic precursor to achieve the consolidation of the printed structure.

**Figure 3:**
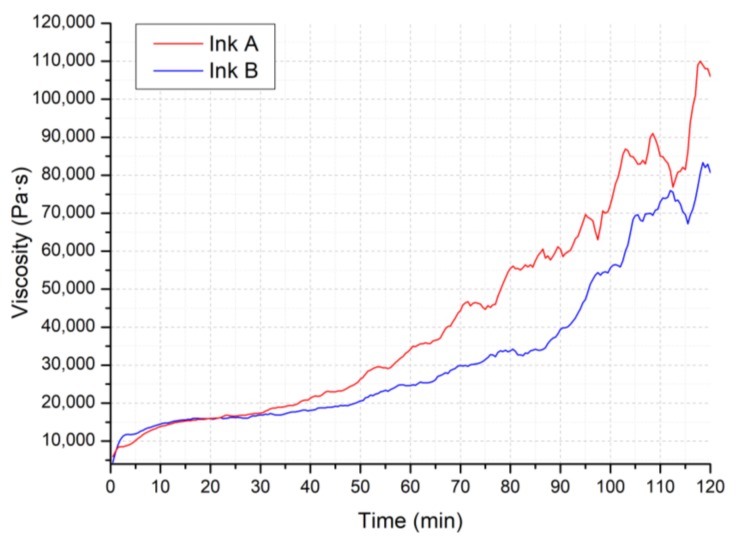
Viscosity η (Pa·s) against time *t* (min) of Ink A and Ink B under constant shear rate γ′ = 0.01 1/s over 2 h.

Figure 3 plots the viscosity η (Pa·s) against time *t* (min) for the two inks; a very low shear rate was applied in order to avoid interfering with the polymer crosslinking over time. The addition of the crosslinking catalyst in the inks is necessary in order to eliminate the softening of the preceramic polymer upon pyrolysis, with concurrent loss of shape for the printed object. However, its limited amount in the formulations allows for a printing window of at least 1 h, during which the viscosity of the inks does not vary significantly, and therefore the rheology tests we carried out were not affected by the ongoing crosslinking reaction.

It was also observed that Ink B had a wider working range compared to Ink A; in fact, the evaporation of the solvent present in a lower amount in Ink B did not affect the viscosity of the ink as much as for Ink A.

**Figure 4:**
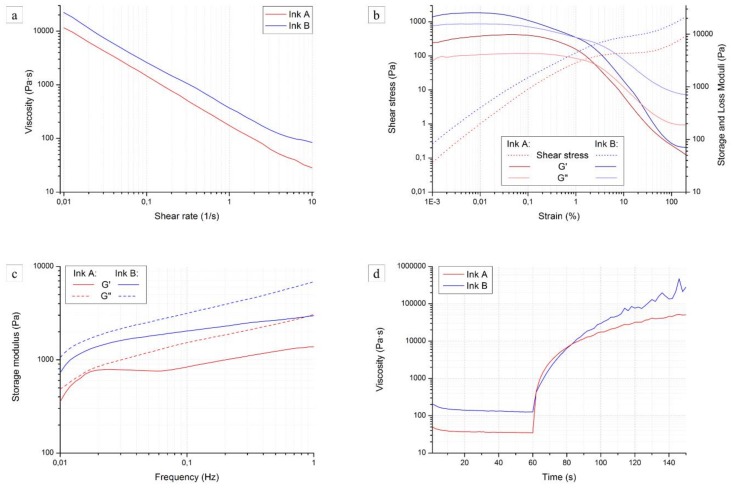
Rheological characterization of Ink A and Ink B: (**a**) viscosity η (Pa·s) against shear rate γ′ (1/s); (**b**) storage modulus G′ (Pa), loss modulus G′′ (Pa), and shear stress τ (Pa) against strain γ (%); (**c**) G′ (Pa) and G′′ (Pa) against frequency; (**d**) η (Pa·s) against time *t* (s).

Figure 4a shows the viscosity curves of the two inks, plotting the viscosity η (Pa·s) against the shear rate γ′ (1/s). Unfortunately, the inks lacked in adhesion to the rheometer plates and tended to be expelled from the side even at low shear rates (above 10 1/s); the measured segments of the viscosity curves did not represent the printing window (the shear rate during actual printing was estimated to be between 50 and 100 1/s), but were significant in determining the behavior of the inks.

Shear thinning behavior was confirmed for Ink A, as suggested by its printing behavior [31]. The destruction of the weak hydrogen bonds bridging the fumed silica particles, together with the intrinsic behavior of the preceramic polymer, are largely responsible of the decrease of viscosity with increasing shear rate. The viscosity of Ink B was about twice as high as that of Ink A and had a similar slope; it can be therefore confirmed that the preceramic polymer can develop a sufficient pseudoplastic behavior by itself when the amount of solvent for its dilution is optimized. The silicone resin employed in this work possesses a branched structure [34] and, when the concentration of the polymer is high enough in the ink, entanglements form and break depending on the shear stress applied, hence the contribution to the pseudoplastic behavior [35].

Dynamic oscillation tests allowed us to assess whether the inks had a transition to low rigidity systems at higher shear stress values. Measuring in oscillatory mode allowed us to partially overcome the adhesion issues previously mentioned.

Figure 4b shows a log-log plot of the storage moduli G′ (Pa), loss moduli G′′ (Pa), and shear stress τ (Pa) of the inks as a function of the strain γ (%). Both materials followed a similar trend:

1. At low to intermediate strain, both G′ and G′′ showed a plateau, with G′ > G′′; the shear stress increased at a constant rate;
2. At high strainer, G′ and G′′ decreased rapidly and intersected; at the same time, the slope of the shear stress decreased;
3. After that, all values stabilized again.

This behavior could be classified as type I (shear thinning) in the large amplitude oscillatory shear (LAOS) system [36], and could originate from the chain orientation or alignment of microstructures along the flow direction, thus reducing the local viscous drag on material elements. As the shear rate is further increased, the flow alignment becomes more complete, and the shear viscosity decreases further. Since these curves did not have a clear transition point, the values of yield stress *τ_y_* were determined at the intersection between the G′ and G′′ curves. As the polymer is not yet crosslinked, chains in the solution are rather short and their orientation can be modified by relatively low shear rates: ~95 Pa for Ink A and ~120 Pa for Ink B.

A slight transition to higher values could be detected for G′, G′′, and τ_y_ curves measured for Ink B, in accordance with the steady rate sweep test results and with the lower amount of solvent present in the ink. Maximum G′ values for Ink A and B were ~9.8 kPa and ~25.6 kPa, respectively. It must be mentioned that the strain sweep test alone cannot guarantee that the inks possess the structure of a (reversible) gel material; therefore, in order to further validate our concept and acquire more information, frequency sweep tests were conducted. 

Figure 4c reports the values for G’ and G” against frequency for the two inks; if we compare them with typical polymers and gels curves, we observe that in the frequency range both inks seem to be beyond the crossover point, with G′ and G′′ increasing slightly over a wide frequency range [37]. This could be caused by a weakly crosslinked structure or by a wide molecular weight distribution of uncrosslinked chains. In any case, the two tests together confirm the desired behavior of the inks. Both inks were beyond the transition region, in the so-called glassy region. Further enhancing of the Bingham pseudoplasticity could lead to an extension of the rubbery plateau to higher frequencies and therefore to an ink better suited for extrusion processes [37].

The most important feature of an ideal ink for direct ink writing is the ability to retain the shape after each filament is deposited, which corresponds to a quick viscosity recovery when no shear stress is applied.

Figure 4d shows the result of the viscosity recovery test conducted on the two inks. From the initial viscosity of ~30 Pa·s due to the applied shear rate, Ink A showed a recovery of more than two orders of magnitude in less than 20 s and tended to a plateau value of ~50 kPa·s. Ink B, on the other hand, started from a slightly higher value (~125 Pa·s) and showed a more modest recovery: 20 s after the release of the shear rate, the inks possessed a similar viscosity of ~7.5 kPa·s. The quicker recovery seen for Ink A is likely due to the rapid reconstruction of the hydrogen bonds between the silica particles, which occurred faster than the misalignment of the polymer chains. Over longer times, the viscosity mismatch between the two inks was detected again.

These results confirm that both slurries can have an appropriate rheology for DIW, as the increase of viscosity (increase in rigidity of the system) to a sufficient value occurs in a very short period of time, therefore reducing the deformation of printed, and especially unsupported structures. The higher values of viscosity reached by Ink B over time are beneficial for overcoming the collapse of the first layers of the structure during printing, rendering components able to better retain their shape; moreover, the elimination of fumed silica from the slurry would allow such components to be used at higher temperatures.

### 3.2. Filaments Characterization

**Figure 5:**
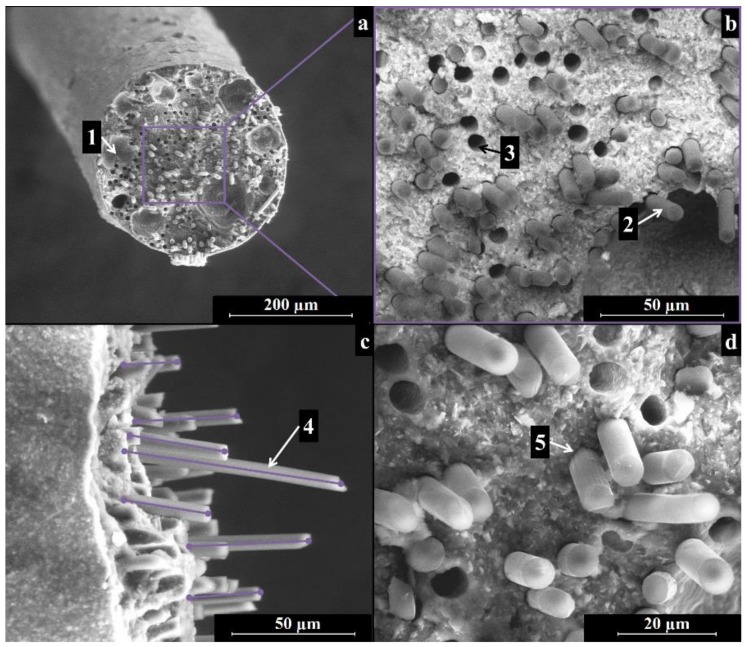
SEM images of a pyrolyzed filament produced with Ink B using a 410-µm nozzle: (**a**) an overview of the cross-section; (**b**) homogeneous fiber and filler distribution in the filament; (**c**) fiber alignment and pull out; (**d**) close up of the interface between fibers and matrix. Legend: (1) air voids; (2) carbon fibers; (3) voids left by carbon fibers pulled-out during fracture; (4) fiber pull-out; (5) interface between matrix and fibers.

**Figure 6:**
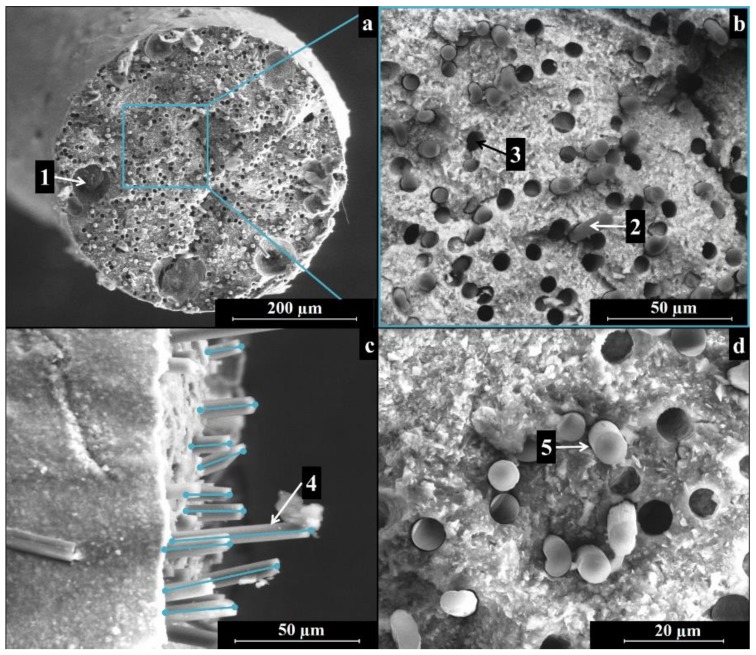
SEM images of a pyrolyzed filament produced with Ink B using a 580-µm nozzle: (**a**) an overview of the cross-section; (**b**) homogeneous fiber and filler distribution in the filament; (**c**) fiber alignment and pull out; (**d**) close up of the interface between fibers and matrix. Legend: (1) air voids; (2) carbon fibers; (3) voids left by carbon fibers pulled-out during fracture; (4) fiber pull-out; (5) interface between matrix and fibers.

Figure 5 and Figure 6 show the cross-section of pyrolyzed filaments printed with 410-µm and 580-µm tips, respectively. The as-printed filaments had a diameter of ~380 µm and ~560 µm, for samples printed with the smaller and larger nozzles, with a linear shrinkage of ~7% and of ~3%, respectively. The difference in shrinkage is likely due to gravity acting on the filaments upon extrusion, suggesting that the evaporation of the solvent did not lead to significant shrinkage. After ceramization, the filaments printed with the smaller tip possessed a diameter of ~310 µm; filaments extruded through the bigger tip possessed a final diameter of ~460 µm. The linear shrinkage upon pyrolysis was therefore ~18% for both inks. In our previous work, the linear shrinkage of printed components was almost negligible (~2%) [31]. The printed three-dimensional (3D) network structure, as well as the presence of fumed silica in that ink, might have been responsible for the limited shrinkage observed.

The ceramized filaments in Figure 5a and Figure 6a were dense and showed no cracks, while the limited presence of some cavities in the cross-section, with a size in the range of ~30 to 100 µm, could derive from air trapped during the preparation of the ink or during the loading of the ink into the extruder. A more efficient mixing approach, using a planetary mixer, will be used in further experiments.

Figure 5b and Figure 6b depict cross-sections of the samples, employing a detector for backscattered electrons. The contrast in the image reflects the different compositions of the matrix and the reinforcement and thus highlights the position of the carbon fibers. The fibers appeared to be well dispersed in the matrix and randomly distributed, indicating that the different components (polymer, SiC powders, and C fibers) were suitably dispersed during the preparation of the ink. 

A close up of the fracture surface shows the good adhesion of the ceramic matrix to the carbon fibers (see Figure 5c and Figure 6c), as well as the pull-out of the fibers in the range of ~10 to 50 µm (see Figure 5c and Figure 6c).

### 3.3. Four-Point Bending Test

**Figure 7:**
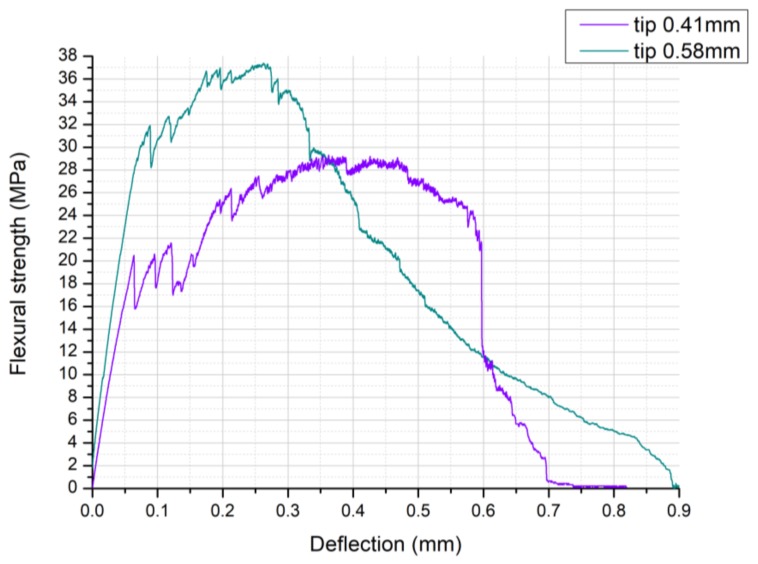
Flexural strength *σ_f_* (MPa) against deflection (mm) for pyrolyzed filaments printed by extruding Ink B through a 410-µm and a 580-µm nozzle.

Figure 7 shows typical strength-deflection curves of filaments printed with the 410-µm and 580-µm tips. According to Equation (5), the flexural strength of the thinner filaments (printed with the 410-µm tip) was 31.7 ± 6.8 MPa; thicker filaments (printed with the 580-µm tip) had a flexural strength of 36.4 ± 4.5 MPa.

The relatively high standard deviation of the measured values was mainly due to irregularities of the filaments, such as surface roughness, distortion, and variations in the cross-section, which could not be eliminated before testing. Some of these defects were caused by geometric irregularities of the tips used for printing and the presence of carbon fibers at the surface of the filaments. If SiC powder agglomerates are present in the ink, during extrusion the flow is not constant and can lead to deformation of the printed part. Warping can also be caused during pyrolysis, because of differences in temperature along the filaments or of a non-completely homogeneous distribution of the preceramic polymer in the matrix. Therefore, at this stage of the investigation no statistically relevant differences between the flexural strength of the two kinds of filament could be detected.

The presence of fibers increased the filament toughness, which could deflect consistently before breaking. This is particularly visible for the thinner filaments, whose maximum deflection could be twice as big as the filament diameter. Thicker filaments already possessed higher rigidity, proven by the higher slope of the first segment of the corresponding curve; therefore, their deflection upon breaking was more moderate.

After the first, linear elastic region, the curves show the presence of several partial, non-catastrophic failures of the filaments (sudden drops of the stress in the curve). These can be interpreted as subsequent fracture of the filament matrix, whose pieces were still kept together by the carbon fibers. In fact, as discussed before, SEM pictures of the fracture surface of the filaments showed the occurrence of pull-out of the fibers (see Figure 5c and Figure 6c). Moreover, the strength-deflection curves themselves show that graceful, non-catastrophic failure occurred in the samples, as demonstrated by the continuing deformation before failure after the maximum stress was reached. As some air voids were contained in the body of the filaments (see Figure 5a and Figure 6a), higher strength values could potentially be expected with a more efficient mixing and degassing of the ink. 

## 4. Conclusions

Inks based on a preceramic polymer, SiC powder, and chopped carbon fibers were developed for the production of CMCs through DIW and subsequent pyrolysis.

The preceramic polymer accomplishes multiple functions:

- It forms a unique SiOC glassy matrix, which can only be synthesized through a molecular route;
- It allows tailoring of the ink rheology thanks to its intrinsic pseudo-plasticity.

An adequate concentration of the polymer alone was proven to be effective for achieving a Bingham shear thinning behavior and fast viscosity recovery after the application of shear stress. 

CMC filaments produced via DIW and pyrolysis at 1000 °C were tested under a four-point bending test in order to understand their mechanism of failure, showing a mean flexural strength above 30 MPa.

SEM images of the filaments sections after fracture highlighted:

- A dense, crack-free matrix in which the carbon fibers (and the SiC powder) appear homogeneously distributed;
- Affective alignment of the fibers along the filament extrusion direction;
- Fiber pull-out at the fracture surface;
- Moreover, the addition of the carbon fibers provided multiple benefits: for example, they increased the toughness of the filaments, which could deflect consistently before breaking, and they caused graceful, non-catastrophic failure after the maximum stress was reached.

These findings reveal the potential to produce anisotropic structures with higher toughness and strength in specific directions or sections of the printed component, thanks to the fiber alignment provided by the DIW process.

## Figures and Tables

**Table 1:** Ink formulations.

Ink	Isopropyl Alcohol (g)	MK (g)	SiC (g)	Carbon Fibers (g)	Fumed Silica (g)	Crosslinker GF91 (mL)	Dispersant BYK430 (mL)
**Ink A**	9	21	17.85	5.35	2.68	0.1	1.15
**Ink B**	9 (before evaporation)	21	14.88	5.95	/	0.1	0.75

## Nomenclature

AM	additive manufacturing
c	distance from the neutral axis (mm)
CMC	ceramic matrix composite
γ	strain (%)
γ′	shear rate (1/s)
*d*	diameter of the tested filament (mm)
DIW	direct ink writing
EBC	environmental barrier coating
*F*	maximum load (N)
FS	fumed silica
G′	storage modulus (Pa)
G′′	loss modulus (Pa)
η	viscosity (Pa·s)
*I*	moment of inertia (mm^4^)
*l*	loading span (mm)
*L*	support span (mm)
LAOS	large amplitude oscillatory shear
LPI	liquid polymer infiltration
M	applied moment (N·mm)
MK	poly(methyl-silsequioxane) Silres MK (Wacker Chemie AG, Nünchritz, DE)
PDC	polymer-derived ceramics
PIP	polymer infiltration and pyrolysis
SEM	scanning electron microscopy
$\sigma_{f}$	flexural strength (MPa)
*t*	time (min)
*τ*	shear stress (Pa)
*τ_y_*	yield stress (Pa)
